# Bacterial and viral infections among adults hospitalized with COVID‐19, COVID‐NET, 14 states, March 2020–April 2022

**DOI:** 10.1111/irv.13107

**Published:** 2023-03-02

**Authors:** Melisa M. Shah, Kadam Patel, Jennifer Milucky, Christopher A. Taylor, Arthur Reingold, Isaac Armistead, James Meek, Evan J. Anderson, Andy Weigel, Libby Reeg, Kathryn Como‐Sabetti, Susan L. Ropp, Alison Muse, Sophrena Bushey, Eli Shiltz, Melissa Sutton, H. Keipp Talbot, Ryan Chatelain, Fiona P. Havers, the CDC COVID‐NET Surveillance Team

**Affiliations:** ^1^ Epidemic Intelligence Service Centers for Disease Control and Prevention Atlanta Georgia USA; ^2^ COVID‐19 Emergency Response Team Centers for Disease Control and Prevention Atlanta Georgia USA; ^3^ General Dynamics Information Technology Atlanta Georgia USA; ^4^ Coronavirus Disease 2019–Associated Hospitalization Surveillance Network, Division for Viral Diseases, National Center for Immunization and Respiratory Diseases Centers for Disease Control and Prevention Atlanta Georgia USA; ^5^ California Emerging Infections Program Oakland California USA; ^6^ University of California, Berkely Berkely California USA; ^7^ Colorado Department of Public Health & Environment Denver Colorado USA; ^8^ Connecticut Emerging Infections Program Yale School of Public Health New Haven Connecticut USA; ^9^ Departments of Medicine and Pediatrics Emory University School of Medicine Atlanta Georgia USA; ^10^ Georgia Emerging Infections Program, Georgia Department of Public Health Atlanta Veterans Affairs Medical Center Atlanta Georgia USA; ^11^ Iowa Department of Health Des Moines Iowa USA; ^12^ Michigan Department of Health and Human Services Lansing Michigan USA; ^13^ Minnesota Department of Health Saint Paul Minnesota USA; ^14^ New Mexico Department of Health Santa Fe New Mexico USA; ^15^ New York State Department of Health Albany New York USA; ^16^ University of Rochester School of Medicine and Dentistry Rochester New York USA; ^17^ Ohio Department of Health Columbus Ohio USA; ^18^ Public Health Division Oregon Health Authority Portland Oregon USA; ^19^ Vanderbilt University Medical Center Nashville Tennessee USA; ^20^ Salt Lake County Health Department Salt Lake City Utah USA; ^21^ US Public Health Service Commissioned Corps Rockville Maryland USA

**Keywords:** bacterial coinfection, COVID‐19, COVID‐NET, SARS‐CoV‐2, viral coinfection

## Abstract

**Background:**

Bacterial and viral infections can occur with SARS‐CoV‐2 infection, but prevalence, risk factors, and associated clinical outcomes are not fully understood.

**Methods:**

We used the Coronavirus Disease 2019‐Associated Hospitalization Surveillance Network (COVID‐NET), a population‐based surveillance system, to investigate the occurrence of bacterial and viral infections among hospitalized adults with laboratory‐confirmed SARS‐CoV‐2 infection between March 2020 and April 2022. Clinician‐driven testing for bacterial pathogens from sputum, deep respiratory, and sterile sites were included. The demographic and clinical features of those with and without bacterial infections were compared. We also describe the prevalence of viral pathogens including respiratory syncytial virus, rhinovirus/enterovirus, influenza, adenovirus, human metapneumovirus, parainfluenza viruses, and non‐SARS‐CoV‐2 endemic coronaviruses.

**Results:**

Among 36 490 hospitalized adults with COVID‐19, 53.3% had bacterial cultures taken within 7 days of admission and 6.0% of these had a clinically relevant bacterial pathogen. After adjustment for demographic factors and co‐morbidities, bacterial infections in patients with COVID‐19 within 7 days of admission were associated with an adjusted relative risk of death 2.3 times that of patients with negative bacterial testing. *Staphylococcus aureus* and Gram‐negative rods were the most frequently isolated bacterial pathogens. Among hospitalized adults with COVID‐19, 2766 (7.6%) were tested for seven virus groups. A non‐SARS‐CoV‐2 virus was identified in 0.9% of tested patients.

**Conclusions:**

Among patients with clinician‐driven testing, 6.0% of adults hospitalized with COVID‐19 were identified to have bacterial coinfections and 0.9% were identified to have viral coinfections; identification of a bacterial coinfection within 7 days of admission was associated with increased mortality.

## 1 BACKGROUND

Coinfections, both bacterial and viral, occur with viral respiratory tract infections and can be associated with increased morbidity and mortality^1^, ^2^ but can also be incidental.^3^ Polymicrobial respiratory infections may stem from compromised mucosal lung structure and altered immune responses after an initial infection.^4^, ^5^ Secondary bacterial infections are known complications of severe influenza infection^6^; however, studies of patients infected with severe acute respiratory syndrome coronavirus 2 (SARS‐CoV‐2) suggest that bacterial coinfection and secondary infections among patients with COVID‐19 are relatively uncommon.^3^, ^7^, ^8^ Population‐based data are limited on the prevalence of bacterial and viral coinfections in adults with COVID‐19.

We used data collected through Coronavirus Disease 2019‐Associated Hospitalization Surveillance Network (COVID‐NET), a large, geographically diverse US population‐based surveillance platform, to investigate the proportion of viral and bacterial infections among hospitalized adults with laboratory‐confirmed COVID‐19. In this study, our objectives were to (1) compare demographic, radiographic and clinical features, and outcomes among those with bacterial infections, (2) characterize the microbial spectrum of bacterial infections, and (3) describe the prevalence of viral infections. Understanding the epidemiology of bacterial and viral infections in adults hospitalized with COVID‐19 and the association with disease severity can inform testing for coinfections and approach to antimicrobial treatment.

## 2 METHODS

### 2.1 Study population

Data were collected through COVID‐NET,^9^ a population‐based surveillance system including more than 250 acute‐care hospitals across 99 counties in 14 states (California, Colorado, Connecticut, Georgia, Iowa, Maryland, Michigan, Minnesota, New Mexico, New York, Ohio, Oregon, Tennessee, and Utah) covering approximately 10% of the US population. COVID‐NET captures laboratory confirmed COVID‐19‐associated hospitalizations, defined as any patient residing in the catchment areas with a positive SARS‐CoV‐2 test during hospitalization or during the 14 days prior to admission. A full case report form is completed on a representative sample of cases stratified by age group and site. For sample selection, random numbers were generated and assigned to each case as previously described.^10^ Sampling weights are assigned based on the probability of selection. Trained staff collect demographic information, signs and symptoms, underlying comorbidities, chest imaging, viral and bacterial testing, and outcomes including need for mechanical ventilation, critical care, and death. Individuals with a positive SARS‐CoV‐2 test were included regardless of reason for admission.

### 2.2 Bacterial pathogens

Adults 18 years or older hospitalized with COVID‐19 during March 2020 and April 2022 and receiving any culture testing within 7 days of admission (including 7 days before or 7 days after) were included in this cross‐sectional analysis of bacterial pathogens. Only pathogens from sterile or respiratory sites were included; cultures from the nasopharynx, urine, or superficial sites (e.g., wound cultures) were excluded. Multiple pathogens were included when present. For individuals with multiple cultures with the same pathogen, results of each bacterial infection and dates of specimen collection were recorded in the case report form. Dates of negative cultures were not recorded. The percent of all sampled cases with any bacterial testing was examined quarterly during the study period.

For individuals with a bacterial infection, the site of culture positivity was classified as sputum, deep respiratory, blood, or other sterile site. Deep respiratory sites included endotracheal aspirate, bronchoalveolar lavage fluid, pleural fluid, and lung tissue. Examples of other sterile sites include cerebrospinal fluid, bone, and peritoneal fluid.

Two physicians trained in infectious diseases reviewed culture information; bacterial infections were defined as clinically relevant organism from a respiratory or sterile site. Individuals categorized as not having a bacterial infection were those who had cultures taken within 7 days of admission (before or after) but tested negative for potentially clinically relevant bacteria. Anaerobes, *Bacillus* species, *Corynebacterium*, and *coagulase‐negative Staphylococcus* (except for *Staphylococcus lugdunensis*) speciated from blood were excluded unless present in multiple cultures collected on separate days. From sputum cultures, *Enterococcus* and *Streptococcus* species (except *Streptococcus pneumoniae*) were excluded. *Coagulase‐negative Staphylococcus* were excluded from sputum and deep respiratory cultures as they are usually commensals or contaminants.

### 2.3 Viral pathogens

Clinician‐directed testing by polymerase chain reaction (PCR) testing results for respiratory syncytial virus (RSV), rhinovirus/enterovirus (RV/EV), influenza (subtypes A, B, or unspecified), adenovirus, human metapneumovirus (HMPV), parainfluenza (serotypes 1–4), and seasonal human coronaviruses (229E, HKU1, NL63, OC43) were also collected from 7 days prior and through 7 days after admission.

### 2.4 Data analysis

For bacterial infections, we examined characteristics of patients with and without culture testing. Among those with testing, we compared characteristics of those with and without detection of bacterial infections within 7 days before or after hospital admission. We used chi‐square testing for categorical variables and Wilcoxon rank sum tests for continuous variables. We then quantified the most common organisms recovered by site. The same pathogen species and site was only counted once from an individual. We examined whether having a clinically relevant bacterial pathogen with SARS‐CoV‐2 infection was associated with worse outcomes including intensive care unit (ICU) admission, receiving invasive mechanical ventilation (IMV), or death during the hospitalization using multivariable logistic regression analysis with generalized estimating equations (GEE) and controlling for age, sex, race and ethnicity, underlying medical conditions, and time period. Variables significant in bivariate analysis and considered to be relevant to the outcome were included. We present adjusted relative risks (RR) with 95% confidence intervals. A sensitivity analysis on the association of bacterial infection and death was completed excluding individuals with first positive culture on the second day of ICU admission or later. For each respiratory virus, we calculated the proportion of individuals testing positive among all hospitalized adults with COVID‐19 who were tested. Due to low number of viral coinfections, additional multivariable analyses were not performed. Analyses were performed using SAS (version 9.4; SAS Institute). COVID‐NET uses a sampling scheme and collects clinical data on a representative sample of hospitalized adults.^11^ All findings are weighted to account for the probability of selection of the sampled patients and adjusted to account for charts with incomplete or missing data. Unweighted counts and weighted percentages are reported except when indicated. The variance estimation was conducted using the Taylor series linearization method.

### 2.5 Ethical review

This activity was reviewed by the Centers for Disease Control and Prevention (CDC) and conducted consistent with applicable federal law and CDC policy (see e.g., 45 C.F.R. part 46.102(l) (2), 21 C.F.R. part 56; 42 U.S.C. §241(d); 5 U.S.C. §552a; 44 U.S.C. §3501 et seq.). When required, participating sites obtained approval from respective state and local institutional review boards.

## 3 RESULTS

**FIGURE 1:**
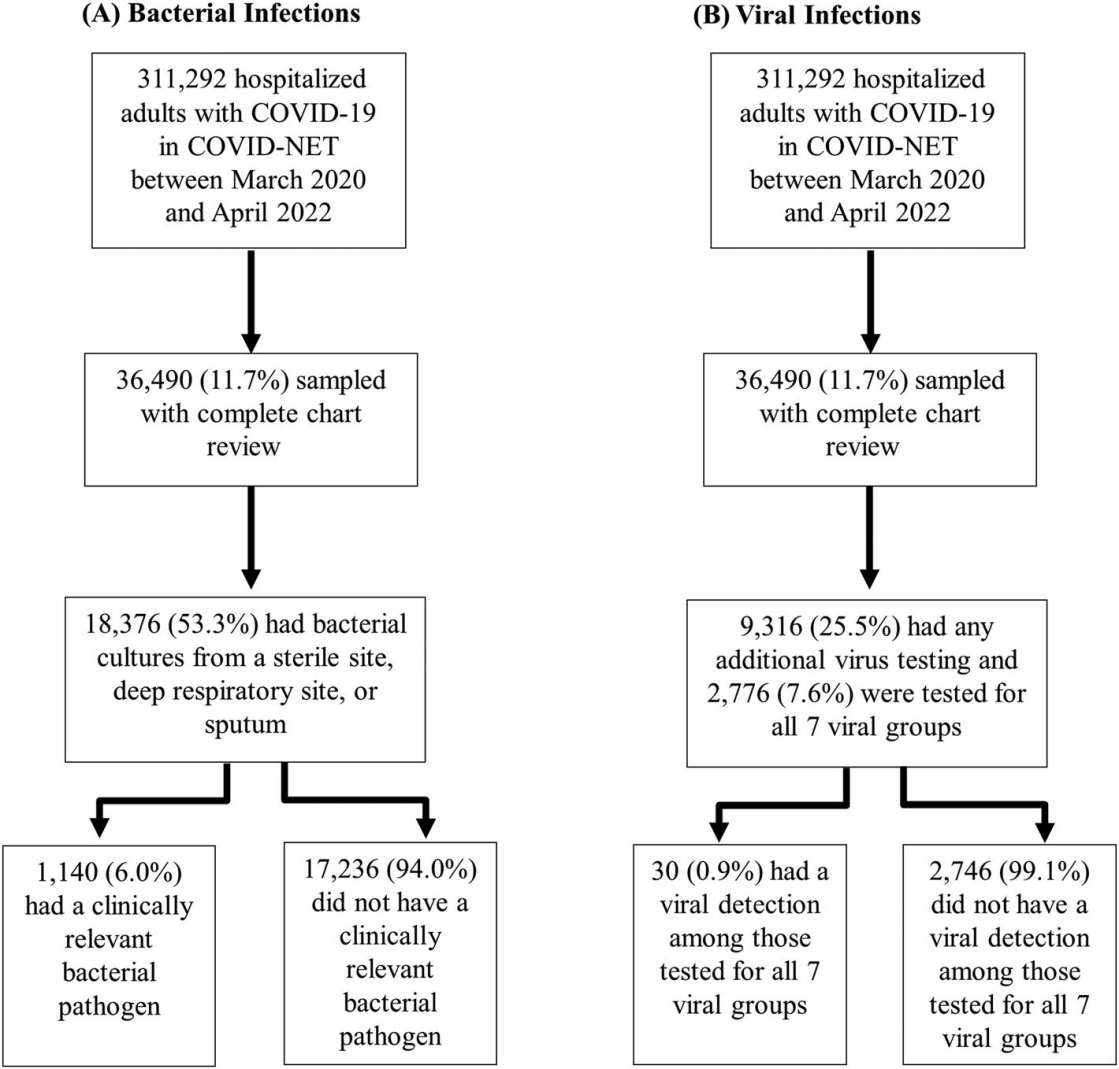
Flowchart of hospitalized adults in COVID‐NET with bacterial and viral infections. Unweighted counts and weighted percentages are reported. The seven viral groups include respiratory syncytial virus, rhinovirus/enterovirus, influenza (subtypes A, B, or unspecified), adenovirus, human metapneumovirus (HMPV), parainfluenza (serotypes 1–4), and common human coronaviruses (229E, HKU1, NL63, OC43).

Between March 2020 to April 2022, there were a total of 311 292 hospitalizations among adults in COVID‐NET. Of those, a representative sample of 36 490 hospitalized adults had a complete medical chart review. Among 36 490 adults hospitalized in the COVID‐NET catchment area during March 2020 and April 2022, 18 376 (53.3%) had bacterial cultures from sputum, deep respiratory, blood, or other sterile sites (Figure 1A). Of these, 1140, (6.0%) had a bacterial pathogen identified. The percent of all sampled cases with bacterial testing ranged from 46.3% to 70.3% when examined quarterly, and culture testing generally decreased over time (Figure S1). Individuals receiving any bacterial testing were more likely to have underlying conditions (92.1% vs. 81.9%, *p* < 0.0001), receive ICU level care (30.4% vs. 11.7%, *p* < 0.0001), and had higher mortality (14.3% vs. 5.1%, *p* < 0.0001) compared to those without bacterial testing (Table S1). Among those with a bacterial infection, individuals had a median of 1 bacterial infection; the median earliest specimen collection date was on the first day of admission (0.9, interquartile range 0.4–5.0). Among 18 376 patients with bacterial cultures performed (Table 1) within 7 days of admission, sex, age, and race/ethnicity were not significantly different among those with cultures positive for potentially clinically relevant bacteria compared with those with negative cultures. Individuals with bacterial infections were more likely to have underlying medical conditions in the bivariate analysis; 67.3% of those with a bacterial infection had three or more underlying conditions compared with 58.7% of those with bacterial cultures negative for clinically relevant pathogens (*p* = 0.0017) (Table 1). Those with bacterial infections were more likely to have chronic lung disease, diabetes, cardiovascular disease, obesity, gastrointestinal/liver disease, and renal disease compared with those without bacterial infections within 7 days of admission (Table 1). Cough was less commonly reported in those with a bacterial infection compared with those without a bacterial infection (52.5% vs. 62.9%, *p* < 0.0001) (Table 1).

**TABLE 1:** Baseline characteristics of hospitalized adults with COVID‐19 and bacterial testing performed within 7 days of admission, COVID‐NET March 2020**–**April 2022, stratified by presence of bacterial infections. Unweighted counts and weighted percentages are reported.

	Total sampled hospitalized adults with COVID‐19 tested for a bacterial infection (*n* = 18 376)		Clinically relevant bacterial infection within ±7 days of admission (*n* = 1140)		No clinically relevant bacterial infection within first 7 days of admission among those with any bacterial culture performed (*n* = 17 236)		
	*n*	Weighted column % with 95% CI	*n* = 1140	Weighted column % with 95% CI	*n* = 17 236	Weighted column % with 95% CI	*p* value
Sex							0.0793
Male	10 060	54.0 (52.6–55.3)	697	58.2 (53.2–63.1)	9363	53.7 (52.3–55.0)	
Female	8316	46.0 (44.7–47.4)	443	41.8 (36.9–46.8)	7873	46.3 (45.0–47.7)	
Age category							0.6917
18–34 years	1865	7.3 (6.7–8.0)	97	6.0 (4.1–8.6)	1768	7.4 (6.8–8.1)	
35–54 years	5185	23.3 (22.3–24.4)	316	22.9 (19.2–27.0)	4869	23.4 (22.3–24.4)	
55–74 years	7317	42.1 (40.8–43.4)	496	44.0 (39.1–49.0)	6821	42.0 (40.7–43.3)	
≥75 years	4009	27.2 (26.0–28.5)	231	27.0 (22.6–31.8)	3778	27.2 (25.9–28.6)	
Race and ethnicity							0.2816
Non‐Hispanic White	8725	49.0 (47.7–50.3)	548	49.2 (44.1–54.3)	8177	49.0 (47.6–50.4)	
Non‐Hispanic Black	3843	26.8 (25.6–28.0)	239	29.3 (24.6–34.4)	3604	26.6 (25.3–27.8)	
Non‐Hispanic AI/AN	371	1.6 (1.3–1.9)	38	2.1 (1.1–3.5)	333	1.6 (1.3–1.9)	
Asian/PI	1198	5.8 (5.1–6.6)	73	4.0 (2.4–6.2)	1125	5.9 (5.2–6.8)	
Hispanic	3547	16.8 (15.9–17.8)	204	15.4 (12.1–19.1)	3343	16.9 (16.0–17.9)	
Any underlying condition^a^	16 696	92.1 (91.4–92.8)	1068	96.3 (94.6–97.5)	15 628	91.8 (91.1–92.5)	<0.0001
Major underlying conditions^b^	1680	7.9 (7.2–8.6)	72	3.7 (2.5–5.4)	1608	8.2 (7.5–8.9)	0.0017
0	2678	12.3 (11.5–13.1)	74	3.8 (2.5–5.6)	1584	8.3 (7.6–9.1)	
1	3255	16.8 (15.8–17.8)	151	13.2 (9.8–17.2)	2621	13.3 (12.4–14.2)	
2	10 763	63.0 (61.8–64.3)	182	15.7 (12.2–19.6)	3419	19.7 (18.6–20.8)	
3 or more	16 696	92.1 (91.4–92.8)	685	67.3 (62.4–72.0)	9126	58.7 (57.4–60.1)	
Chronic lung disease	9299	54.5 (53.2–55.8)	369	38.0 (32.9–43.3)	4983	30.1 (28.8–31.4)	0.0018
Diabetes	6604	37.9 (36.6–39.2)	464	42.8 (37.8–47.9)	5976	37.7 (36.3–39.1)	0.0476
Blood disorders	669	4.1 (3.5–4.7)	54	5.4 (3.2–8.5)	570	3.9 (3.3–4.6)	0.197
Cardiovascular disease^c^	6242	38.8 (37.5–40.2)	477	50.4 (45.3–55.5)	5839	40.1 (38.7–41.5)	<0.0001
Neurologic disorders	3847	22.8 (21.6–24.0)	295	25.3 (21.1–29.9)	3421	22.4 (21.2–23.7)	0.19
Immunocompromising conditions	2140	13.8 (12.8–14.7)	141	15.0 (11.2–19.5)	1878	13.4 (12.5–14.4)	0.4283
Obesity^d^	8715	48.5 (47.1–49.8)	493	43.7 (38.6–48.9)	8029	49.2 (47.7–50.6)	0.0408
Gastrointestinal/liver disease	1748	10.9 (10.1–11.8)	117	9.6 (7.3–12.5)	1007	6.2 (5.5–6.9)	0.0021
Renal disease	3102	19.5 (18.3–20.6)	229	25.9 (21.4–30.8)	2752	18.8 (17.6–20.1)	0.0014
Rheumatologic/autoimmune disorder	1051	7.3 (6.6–8.1)	68	7.0 (4.6–10.1)	917	7.2 (6.5–8.1)	0.8446
Time period^e^							
Pre‐Delta	14 784	63.2 (61.8–64.5)	846	54.5 (49.4–59.5)	13 938	63.7 (62.3–65.1)	0.0011
Delta	2442	20.6 (19.6–21.6)	176	23.4 (19.4–27.7)	2266	20.4 (19.4–21.5)	
Omicron	1150	16.3 (15.0–17.6)	118	22.2 (17.6–27.3)	1032	15.9 (14.6–17.3)	
Long‐term care facility residence^f^	2601	13.0 (12.1–13.9)	203	14.9 (11.8–18.5)	2339	12.8 (11.9–13.8)	0.2019
Symptoms							
Cough	11 855	62.3 (61.0–63.6)	598	52.5 (47.5–57.5)	11 257	62.9 (61.6–64.3)	<0.0001
Shortness of breath	12 404	65.4 (64.1–66.7)	733	58.8 (53.6–63.8)	11 671	65.8 (64.5–67.2)	0.0054
Congestion	1855	10.6 (9.9–11.4)	89	9.4 (6.9–12.5)	1766	10.7 (9.9–11.5)	0.3855
Wheezing	802	4.6 (4.1–5.1)	51	4.2 (2.7–6.3)	751	4.6 (4.1–5.2)	0.6688
Hemoptysis	271	1.2 (0.9–1.4)	25	2.4 (1.0–4.8)	246	1.1 (0.9–1.3)	0.0283
Chest X‐ray							
Abnormal chest X‐ray	14 735	84.5 (83.5–85.5)	965	88.7 (85.5–91.4)	13 770	84.3 (83.2–85.3)	0.0101
Consolidation	1401	9.1 (8.3–10.0)	125	13.1 (9.9–17.0)	1276	8.8 (8.0–9.7)	0.006
Lobar infiltrate	14 735	84.5 (83.5–85.5)	965	88.7 (85.5–91.4)	13 770	84.3 (83.2–85.3)	0.0101
Outcomes							
Intensive care required	6459	30.4 (29.2–31.5)	747	60.0 (54.9–64.9)	5712	28.5 (27.3–29.7)	<0.0001
Mechanical ventilation	3627	17.5 (16.6–18.5)	594	47.6 (42.6–52.6)	3033	15.6 (14.7–16.6)	<0.0001
Death	2535	14.3 (13.4–15.3)	329	31.7 (27.2–36.5)	2206	13.2 (12.3–14.1)	<0.0001

*Note*: *N* in each cell represents unweighted frequency or numerator and *N* on top row represents denominator. % is prevalence weighted for sampling and non‐response.

Abbreviations: AI/AN, American Indian/Alaska Native; PI, Pacific Islander.

^a^ Any underlying conditions include a condition from one of the following major underlying condition categories (see below).

^b^ Major underlying conditions include chronic lung disease including asthma; chronic metabolic disease including diabetes; blood disorders/hemoglobinopathies; cardiovascular disease (excluding hypertension); neurologic disorder; immunocompromised condition; renal disease; any obesity; postpartum; gastrointestinal or liver disease; rheumatologic, autoimmune, or inflammatory conditions; other conditions. For definition of major conditions, see Table S4.

^c^ Cardiovascular disease excludes hypertension.

^d^ Obesity is defined as calculated body mass index (BMI) ≥ 30 kg/m^2^, and if BMI is missing, by International Classification of Diseases discharge diagnosis codes.

^e^ Pre‐Delta: March 2020**–**June 2021, Delta: July 2021**–**December 18, 2021, Omicron: December 19, 2021**–**April 2022.

^f^ Long‐term care facility residence includes nursing home/skilled nursing facility, alcohol/drug abuse treatment center, other rehabilitation facility, assisted living/residential care, group/retirement homes, long‐term care facility (LTCF), long‐term acute care hospital (LTACH), or any other psychiatric facility.

Those with bacterial infections were more likely to receive intensive care during hospitalization (60.0% vs. 28.5%, *p* < 0.0001) and to require mechanical ventilation (47.6% vs. 15.6%, *p* < 0.0001) compared with those who had bacterial testing performed and were negative for clinically relevant pathogens. In‐hospital death occurred in 329 (31.7%) of those with bacterial infections compared with 13.2% of those without bacterial infections in bivariate analysis (*p* < 0.0001). Both a respiratory sample culture and blood culture were positive in 9.4% of those with in‐hospital death. After controlling for demographic factors, underlying medical conditions, and time period, adults with COVID‐19 who had bacterial infections within 7 days of admission had 2.28 (95% CI 1.87–2.79) times increased risk for death compared with those with negative bacterial cultures within 7 days of admission (Table 2). After excluding 116 individuals with first positive bacterial culture on the second day of ICU admission or later, those with bacterial infections had 1.81 (95% CI 1.51–2.18) times increased risk for death. Those with a clinically relevant pathogen identified were associated with an increased need for intensive care (RR 2.11 95% CI 1.95–2.23) and mechanical ventilation (RR 3.04 95% CI 2.74–3.37) (Tables S2 and S3).

**TABLE 2:** Adjusted relative risk for death among adults hospitalized with COVID‐19 who had bacterial testing performed within 7 days of admission, COVID‐NET March 2020**–**April 2022.

	Adjusted relative risk for death (95% CI) *n* = 16 383	*p* value
Bacterial infection in a respiratory or sterile site within 7 days of hospital admission	2.28 (1.87, 2.79)	<0.0001
Sex		
Male	1.19 (0.99, 1.43)	0.0618
Female	Ref	Ref
Age category		
18–34 years	Ref	Ref
35–54 years	1.7 (1.16, 2.49)	0.0069
55–74 years	4.01 (2.44, 6.52)	<0.0001
75 years or more	5.68 (3.44, 9.36)	<0.0001
Race/ethnicity		
Non‐Hispanic White	Ref	Ref
Non‐Hispanic Black	0.99 (0.88, 1.12)	0.9123
Non‐Hispanic AI/AN	1.16 (0.91, 1.49)	0.2297
Asian/PI	1.23 (1.07, 1.49)	0.0031
Hispanic	1.41 (1.13, 1.76)	0.0028
Chronic lung disease	0.95 (0.81, 1.13)	0.5763
Diabetes	1.06 (0.94, 1.2)	0.3423
Cardiovascular disease	1.25 (1.07, 1.47)	0.0053
Obesity	1.09 (0.94, 1.27)	0.2428
Gastrointestinal/liver disease	0.83 (0.64, 1.07)	0.142
Renal disease	1.29 (1.17, 1.42)	<0.0001
Time period^a^		
Pre‐Delta	Ref	Ref
Delta	1.39 (1.24, 1.55)	<0.0001
Omicron	0.70 (0.53, 0.92)	0.0103

Abbreviations: AI/AN, American Indian/Alaska Native; PI, Pacific Islander.

^a^ Pre‐Delta: March 2020**–**June 2021, Delta: July 2021**–**December 182 021, Omicron: December 19, 2021**–**April 2022.

**FIGURE 2:**
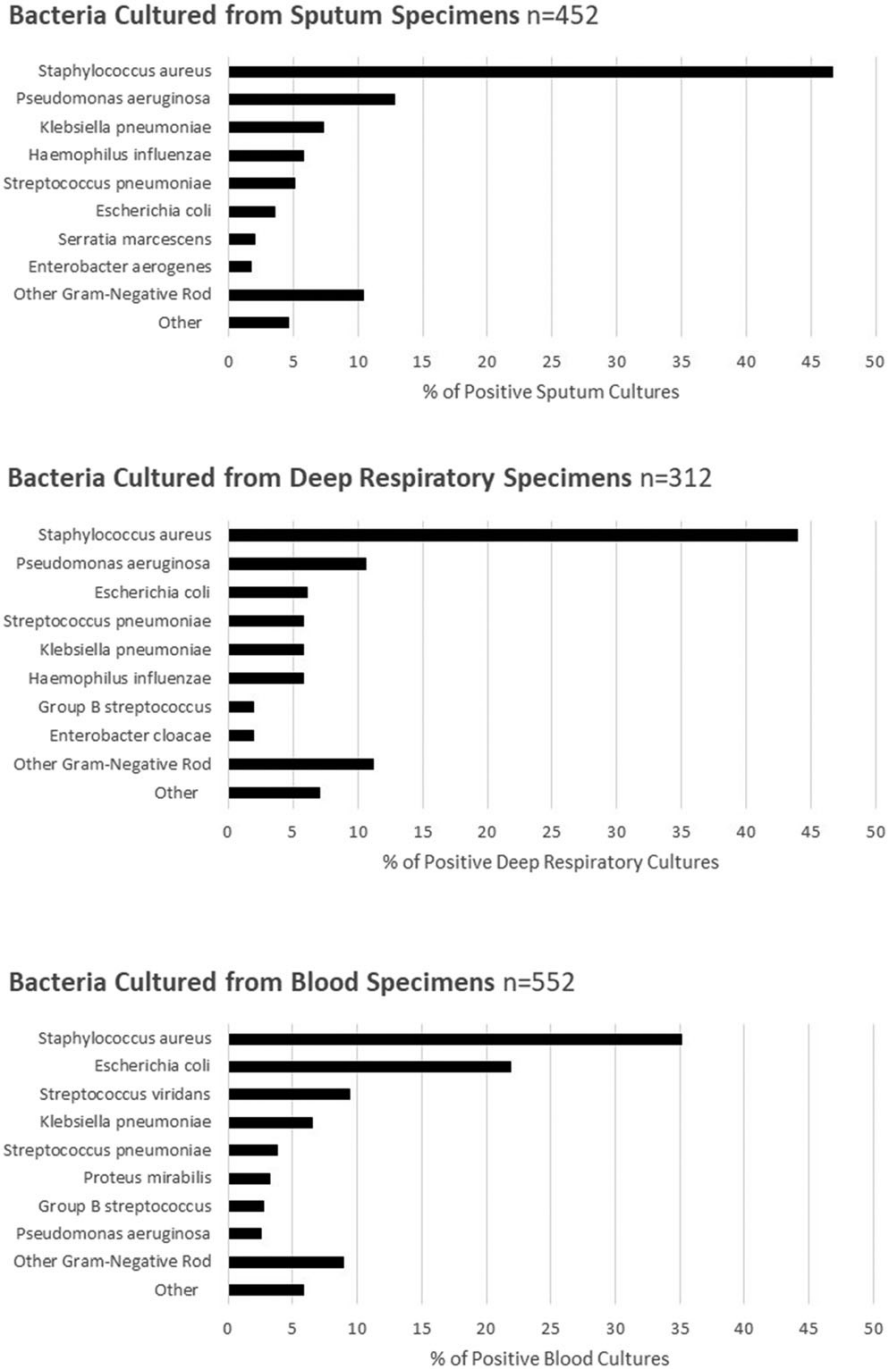
Bacterial cultures from hospitalized sampled adults with COVID‐19 with bacterial pathogens detected in sputum, deep respiratory, or blood cultures within 7 days of admission from Coronavirus Disease 2019‐Associated Hospitalization Surveillance Network (COVID‐NET) from March 2020 to April 2022. This figure includes 1408 bacterial cultures with a clinically relevant organism from 1066 individuals. Deep respiratory sites include endotracheal aspirate, bronchoalveolar lavage fluid, pleural fluid, and lung tissue. Unweighted counts and percentages are reported in this figure.

Of 1140 patients with a bacterial infection, 35.4% had a positive sputum culture, 44.0% had a positive blood culture, 23.4% had a positive deep respiratory culture, and 3.2% had a positive culture from another sterile site. Among those with positive sputum bacterial cultures, 46.7% were *Staphylococcus aureus*. Gram‐negative rods were the next most common organisms recovered in sputum, with *Pseudomonas aeruginosa* (12.8%) and *Klebsiella pneumoniae* (7.3%) most frequently identified (Figure 2). *S. pneumoniae* comprised 5.1% of positive sputum cultures. Among 312 deep respiratory cultures, the most common organism reported was *S. aureus* (43.9%) followed by Gram‐negative rods including *P. aeruginosa* (10.6%), *Escherichia coli* (6.1%), and *K. pneumoniae* (5.8%), (Figure 2). In blood cultures, *S. aureus* (35.1%) and *E. coli* (21.9%) were the most common bacteria isolated. Among the 329 (31.7%) who died, 47.5% had a positive blood culture, 36.6% had a positive sputum culture, 27.7% had a positive deep respiratory culture, and 9.3% had both a positive blood and respiratory culture. In the subset of hospitalized adults (*n* = 329) with COVID‐19 who died, *S. aureus* (46.3%) and *E. coli* (17.0%) were the most frequent pathogens in blood, *S. aureus* (51.7%) and *P. aeruginosa* (12.4%) in sputum specimens, and *S. aureus* (40.2%) and *P. aeruginosa* (17.2%) in deep respiratory specimens.

Among COVID‐19 patients with a complete chart review, 9316 (25.5%) had testing for other respiratory viruses (Figure 1). Of the 9181 individuals tested for influenza (Table 3), 12 (0.1%) were positive; 5639 were tested for RSV, among whom 3 (0.01%) were positive. Of 2766 individuals tested for the seven main viral respiratory groups (parainfluenza, adenovirus, HMPV, RV/EV, RSV, common human coronaviruses, and influenza), another virus was detected in 30 (0.9%). The most commonly detected virus was RV/EV (0.6%) (Table 3). There were two hospitalized adults with COVID‐19, another respiratory virus, and a bacterial pathogen within 7 days.

**TABLE 3:** Viral detections by polymerase chain reaction (PCR) in hospitalized adults with COVID‐19 within 7 days of admission from COVID‐NET March 2020**–**April 2022. Viral groups include polymerase chain reaction (PCR) for influenza (subtypes A, B, or unspecified), rhinovirus/enterovirus, respiratory syncytial virus, adenovirus, human metapneumovirus, parainfluenza (serotypes 1–4), and common human coronaviruses (229E, HKU1, NL63, OC43).

Viral infection by PCR testing	Detections/tested (%)
Rhinovirus/enterovirus	15/3174 (0.6%)
Human metapneumovirus	6/3213 (0.3%)
Parainfluenza	5/3239 (0.3%)
Adenovirus	4/3237 (0.3%)
Common coronavirus type (4 types)	3/2990 (0.3%)
Influenza	12/9181 (0.1%)
Respiratory syncytial virus	3/5639 (0.01%)
Any respiratory co‐detection	30/2776 (0.9%)

## 4 DISCUSSION

Using data from a representative sample of 36 490 cases from 311 292 hospitalized adults with laboratory‐confirmed SARS‐CoV‐2 infection, 6.0% of those with bacterial cultures had evidence of infection with a potentially clinically relevant bacteria within 7 days of admission. Although bacterial infections were identified relatively infrequently during the first week of hospitalizations, among those with bacterial infections identified, nearly one third experienced in‐hospital death. The risk of death was over twofold greater with a detected bacterial infection and COVID‐19 compared with those without evidence of bacterial infections among those subjected to bacterial testing. For clinicians treating patients with COVID‐19 and bacterial infections, understanding that bacterial infections are associated with severe outcomes can inform clinical care.

Our results are consistent with other studies of patients infected with SARS‐CoV‐2 that suggest that bacterial coinfection and secondary infections among patients with COVID‐19 are relatively uncommon^8^ when compared with bacterial coinfections observed with influenza and RSV.^7^, ^12^, ^13^ These findings build upon other reports, including meta‐analyses,^7^, ^8^ which suggest that bacterial infections with COVID‐19 occur in a small subset. Antimicrobial use among hospitalized COVID‐19 early in the pandemic was high, with a meta‐analysis showing 65% prevalence of antibiotic use from studies in the U.S.^14^ In settings with minimal empiric antimicrobial use such as that reported from a Japanese hospital, bacterial coinfections with SARS‐CoV‐2 were also uncommon.^15^ Similarly, viral coinfections are also relatively infrequent; one US study reported 4.2% of patients of all ages attending inpatient or outpatient care with COVID‐19 had viral codetections.^16^

A large population‐based analysis from the United Kingdom concluded that bacterial coinfection were not associated with inpatient death^13^ while other studies have reported an association.^17^ Our analysis suggests an association between bacterial infection and increased death among hospitalized patients with COVID‐19 who receive bacterial testing. More severe COVID‐19 could lead to increased instrumentation and ports of entry for bacterial infections associated with ICU admission. However, even after excluding those with first positive cultures 2 days or more after ICU admission, an increased risk for death remained. Further studies are needed to untangle these interactions between pathogens and host to determine the role of bacterial infections in the natural history of COVID‐19 and to understand to what extent bacterial infections may drive severe COVID‐19 outcomes.

There are several limitations to this analysis. These findings may not be generalizable to all US adults hospitalized with COVID‐19, and testing practices vary across clinicians and facilities. Additionally, the percent of adults with COVID‐19 receiving bacterial testing changed over time. Assigning clinical significance to specific pathogens is difficult, and without full clinical context, classification of pathogens as clinically relevant may be inaccurate. Detection of viral pathogens by PCR or bacterial pathogens by culture may not indicate active infection or disease. The COVID‐NET platform only collects bacterial pathogens within 7 days of admission, making it difficult to describe the burden of secondary or hospital‐acquired infections over the full course of hospitalization and limits the ability to compare with other studies. The group of patients with negative culture testing did not have dates of culture testing limiting comparison of specific time points. Since the temporal sequence of the presence of multiple pathogens was not available, we are unable to attribute causality of outcomes. The population that had bacterial cultures performed were likely more ill or inherently different from patients with COVID‐19 who did not have bacterial cultures performed which may introduce confounding by indication, limiting the generalizability of the findings to all hospitalized adults with COVID‐19. Incidental COVID‐19 admissions were included in the analysis and may contribute to observed differences.

Although bacterial infections in COVID‐19 patients are relatively infrequent, the presence of bacterial infections is associated with significantly increased disease severity, including increased mortality. As SARS‐CoV‐2 continues to circulate and individuals continue to be hospitalized for COVID‐19, understanding risk factors for bacterial infections and associated outcomes can help guide clinicians in providing optimal care.

## AUTHOR CONTRIBUTION

**Melisa M. Shah:** Conceptualization; investigation; methodology; project administration; visualization; writing‐original draft. **Kadam Patel:** Conceptualization; validation; data curation; formal analysis; writing‐review and editing. **Jennifer Milucky:** Conceptualization; methodology; project administration; writing‐review and editing. **Christopher A. Taylor:** Conceptualization; methodology; project administration; writing‐review and editing. **Arthur Reingold:** Writing‐review and editing. **Isaac Armistead:** Writing‐review and editing. **James Meek:** Writing‐review and editing. **Evan J. Anderson:** Writing‐review and editing. **Andy Weigel:** Writing‐review and editing. **Libby Reeg:** Writing‐review and editing. **Kathryn Como‐Sabetti**: Writing‐review and editing. **Susan L. Ropp:** Writing‐review and editing. **Alison Muse:** Writing‐review and editing. **Sophrena Bushey:** Writing‐review and editing. **Eli Shiltz:** Writing‐review and editing. **Melissa Sutton:** Writing‐review and editing. **H. Keipp Talbot:** Writing‐review and editing. **Ryan Chatelain:** Writing‐review and editing. **Fiona P. Havers:** Conceptualization; investigation; methodology; project administration; visualization; writing‐review and editing.

## CONFLICT OF INTEREST STATEMENT

Ms. Leegwater and Ms. Reeg report grants from Michigan Department of Health and Human Services during the conduct of the study. Dr. Anderson reports grants from Pfizer, Merck, PaxVax, Micron, Sanofi‐Pasteur, Janssen, MedImmune, and GSK. Dr. Anderson reports personal fees from Sanofi‐Pasteur, Pfizer, Medscape, Kentucky Bioprocessing, Inc, Sanofi‐Pasteur, Janssen, GSK, WCG and ACI Clinical, and Moderna outside the submitted work. His institution has also received funding from NIH to conduct clinical trials of Moderna and Janssen COVID‐19 vaccines. Mr. Weigel reports grants from CDC/CSTE Cooperative Agreement, during the conduct of the study and grants from CDC/CSTE outside the submitted work. Mr. Teno reports grants from CDC/CSTE Cooperative Agreement during the conduct of the study and grants from CDC/CSTE outside the submitted work. Mrs. Billing reports grants from Council of State and Territorial Epidemiologists (CSTE) during the conduct of the study and grants from Centers for Disease Control and Prevention (CDC) outside the submitted work. Mr. Meek reports grants from CDC during the conduct of the study. Dr. Schaffner reports grants from CDC during the conduct of the study. Dr. Sutton reports grants from CDC Emerging Infections Program during the conduct of the study. Dr. Talbot reports grants from Centers for Disease Control and Prevention, during the conduct of the study. Ms. Yousey‐Hindes reports grants from CDC during the conduct of the study.

## Supporting information


**Table S1:** Baseline characteristics of hospitalized adults with COVID‐19 stratified by presence of bacterial culture testing, COVID‐NET March 2020**–**April 2022
**Table S2:** Adjusted relative risk for intensive care among hospitalized adults with COVID‐19 who had bacterial testing performed within 7 days of admission, COVID‐NET March 2020**–**April 2022
**Table S3:** Adjusted relative risk for mechanical ventilation among hospitalized adults with COVID‐19 who had bacterial testing performed within 7 days of admission, COVID‐NET March 2020**–**April 2022
**Table S4:** Underlying Condition Categories
**Figure S1:** Percent of sampled cases with bacterial culture testing done quarterly over time, COVID‐NET March 2020**–**April 2022

## Data Availability

The data that support the findings of this study are available from the corresponding author upon reasonable request.
