# Supplementary material for: Bacterial and viral infections among adults hospitalized with COVID‐19, COVID‐NET, 14 states, March 2020–April 2022
Source: Influenza Other Respir Viruses. 2023 Mar 2;17(3):e13107. doi: 10.1111/irv.13107 (PMC9981874; doi:10.1111/irv.13107)
Supplement: Supplementary file 1 — Table S1: Baseline characteristics of hospitalized adults with COVID‐19 stratified by presence of bacterial culture testing, COVID‐NET March 2020–April 2022 Table S2: Adjusted relative risk for intensive care among hospitalized adults with COVID‐19 who had bacterial testing performed within 7 days of admission, COVID‐NET March 2020–April 2022 Table S3: Adjusted relative risk for mechanical ventilation among hospitalized adults with COVID‐19 who had bacterial testing performed within 7 days of admission, COVID‐NET March 2020–April 2022 Table S4: Underlying Condition Categories Figure S1: Percent of sampled cases with bacterial culture testing done quarterly over time, COVID‐NET March 2020–April 2022 [file IRV-17-e13107-s001.docx]

**Supplemental Tables and Figures**

**Table S1:** Baseline characteristics of hospitalized adults with COVID-19 stratified by presence of bacterial culture testing, COVID-NET March 2020**–**April 2022

|  | Total sampled hospitalized adults with COVID-19  (n=36,490) | | Bacterial testing performed among hospitalized adults with COVID-19 within 7 days of admission (n=18,376) | | No bacterial testing performed among hospitalized adults with COVID-19 within 7 days of admission (n=18,114) | |  |
| --- | --- | --- | --- | --- | --- | --- | --- |
|  | n | Weight % with 95% CI | n | Weighted column % with 95% CI | n | Weighted column % with 95% CI | p-value |
| Sex |  |  |  |  |  |  | <.0001 |
| Male | 18,366 | 49.9 (49.0-50.8) | 10,060 | 54.0 (52.6-55.3) | 8,306 | 45.4 (44.1-46.7) |  |
| Female | 18,124 | 50.1 (49.2-51.0) | 8,316 | 46.0 (44.7-47.4) | 9,808 | 54.6 (53.3-55.9) |  |
| Age category |  |  |  |  |  |  | <.0001 |
| 18-34 years | 5,720 | 12.1 (11.5-12.7) | 1,865 | 7.3 (6.7-8.0) | 3,855 | 17.4 (16.5-18.4) |  |
| 35-54 years | 10,446 | 24.7 (23.9-25.4) | 5,185 | 23.3 (22.3-24.4) | 5,261 | 26.1 (25.0-27.2) |  |
| 55-74 years | 13,371 | 39.0 (38.1-39.9) | 7,317 | 42.1 (40.8-43.4) | 6,054 | 35.5 (34.3-36.8) |  |
| ≥75 years | 6,953 | 24.2 (23.4-25.1) | 4,009 | 27.2 (26.0-28.5) | 2,944 | 20.9 (19.8-22.1) |  |
| Race/ethnicity |  |  |  |  |  |  | 0.0678 |
| Non-Hispanic White | 17,540 | 49.7 (48.8-50.7) | 8,725 | 49.0 (47.7-50.3) | 8,815 | 50.5 (49.2-51.9) |  |
| Non-Hispanic Black | 7,542 | 26.9 (26.0-27.8) | 3,843 | 26.8 (25.6-28.0) | 3,699 | 27.1 (25.8-28.4) |  |
| Non-Hispanic AI/AN | 664 | 1.5 (1.3-1.7) | 371 | 1.6 (1.3-1.9) | 293 | 1.3 (1.0-1.6) |  |
| Asian/PI | 2,333 | 5.3 (4.9-5.8) | 1,198 | 5.8 (5.1-6.6) | 1,135 | 4.8 (4.2-5.4) |  |
| Hispanic | 7,087 | 16.6 (15.9-17.2) | 3,547 | 16.8 (15.9-17.8) | 3,540 | 16.3 (15.4-17.2) |  |
| Any underlying condition^a^ | 31,080 | 87.3 (86.7-87.8) | 16,696 | 92.1 (91.4-92.8) | 14,384 | 81.9 (80.9-82.8) | <.0001 |
| Major underlying conditions^b^ |  |  |  |  |  |  | <.0001 |
| 0 | 5,410 | 12.7 (12.2-13.3) | 1,680 | 7.9 (7.2-8.6) | 3,730 | 18.1 (17.2-19.1) |  |
| 1 | 5,744 | 13.6 (13.0-14.2) | 2,678 | 12.3 (11.5-13.1) | 3,066 | 15.0 (14.1-15.9) |  |
| 2 | 6,291 | 16.4 (15.7-17.1) | 3,255 | 16.8 (15.8-17.8) | 3,036 | 16.0 (15.0-16.9) |  |
| 3 or more | 19,045 | 57.3 (56.4-58.2) | 10,763 | 63.0 (61.8-64.3) | 8,282 | 50.9 (49.6-52.3) |  |
| Chronic lung disease | 16,862 | 50.8 (49.9-51.7) | 9,299 | 54.5 (53.2-55.8) | 7,563 | 46.7 (45.4-48.0) | <.0001 |
| Diabetes | 11,410 | 33.1 (32.2-34.0) | 6,604 | 37.9 (36.6-39.2) | 4,806 | 27.7 (26.5-29.0) | <.0001 |
| Blood disorders | 1,173 | 3.6 (3.2-4.0) | 669 | 4.1 (3.5-4.7) | 504 | 2.9 (2.5-3.4) | 0.0022 |
| Cardiovascular disease^c^ | 10,958 | 35.2 (34.3-36.2) | 6,242 | 38.8 (37.5-40.2) | 4,716 | 31.2 (30.0-32.5) | <.0001 |
| Neurologic disorders | 6,659 | 19.6 (18.8-20.4) | 3,847 | 22.8 (21.6-24.0) | 2,812 | 16.0 (15.0-17.1) | <.0001 |
| Immunocompromising conditions | 3,504 | 11.6 (11.0-12.3) | 2,140 | 13.8 (12.8-14.7) | 1,364 | 9.2 (8.4-10.1) | <.0001 |
| Obesity^d^ | 16,277 | 48.7 (47.7-49.7) | 8,715 | 48.5 (47.1-49.8) | 7,562 | 49.0 (47.5-50.5) | 0.6213 |
| Gastrointestinal/Liver disease | 3,107 | 10.0 (9.4-10.6) | 1,764 | 10.9 (10.0-11.8) | 1,343 | 9.1 (8.3-9.9) | 0.0028 |
| Renal disease | 5,188 | 17.1 (16.3-17.9) | 3,102 | 19.5 (18.3-20.6) | 2,086 | 14.5 (13.4-15.6) | <.0001 |
| Rheumatologic/Autoimmune disorder | 1,870 | 6.5 (6.0-7.0) | 1,051 | 7.3 (6.6-8.1) | 819 | 5.5 (4.9-6.2) | 0.0004 |
| Time period^e^ |  |  |  |  |  |  |  |
| Pre-delta | 28,601 | 59.7 (58.8-60.7) | 14,784 | 63.2 (61.8-64.5) | 13,817 | 55.9 (54.6-57.3) | <.0001 |
| Delta | 5,165 | 21.7 (20.9-22.4) | 2,442 | 20.6 (19.6-21.6) | 2,723 | 22.9 (21.8-24.0) |  |
| Omicron | 2,724 | 18.6 (17.7-19.5) | 1,150 | 16.3 (15.0-17.6) | 1,574 | 21.2 (19.8-22.6) |  |
| Long term care facility residence^f^ | 4,254 | 10.7 (10.2-11.3) | 2,601 | 13.0 (12.1-13.9) | 1,653 | 8.2 (7.5-9.0) | <.0001 |
| Symptoms |  |  |  |  |  |  |  |
| Cough | 21,060 | 56.3 (55.3-57.2) | 11,855 | 62.3 (61.0-63.6) | 9,205 | 49.6 (48.2-50.9) | <.0001 |
| Shortness of breath | 21,845 | 59.0 (58.0-59.9) | 12,404 | 65.4 (64.1-66.7) | 9,441 | 51.7 (50.4-53.1) | <.0001 |
| Congestion | 3,524 | 10.5 (9.9-11.0) | 1,855 | 10.6 (9.9-11.4) | 1,669 | 10.3 (9.5-11.1) | 0.5538 |
| Wheezing | 1,329 | 4.0 (3.6-4.3) | 802 | 4.6 (4.1-5.1) | 527 | 3.3 (2.8-3.8) | 0.0004 |
| Hemoptysis | 448 | 1.0 (0.9-1.2) | 271 | 1.2 (0.9-1.4) | 177 | 0.9 (0.7-1.2) | 0.1087 |
| Chest x-ray |  |  |  |  |  |  |  |
| Abnormal chest x-ray | 24,527 | 80.1 (79.3-81.0) | 14,735 | 84.5 (83.5-85.5) | 9,792 | 73.9 (72.4-75.3) | <.0001 |
| Consolidation | 2,087 | 8.1 (7.5-8.8) | 1,401 | 9.1 (8.3-10.0) | 686 | 6.5 (5.7-7.5) | <.0001 |
| Lobar infiltrate | 452 | 2.3 (1.9-2.8) | 311 | 2.7 (2.2-3.4) | 141 | 1.7 (1.2-2.2) | 0.0065 |
| Outcomes |  |  |  |  |  |  |  |
| Intensive care required | 8,803 | 21.6 (20.9-22.4) | 6,459 | 30.4 (29.2-31.5) | 2,344 | 11.7 (10.9-12.6) | <.0001 |
| Mechanical ventilation | 4,522 | 11.7 (11.1-12.3) | 3,627 | 17.5 (16.6-18.5) | 895 | 5.1 (4.6-5.7) | <.0001 |
| Death | 3,269 | 10.0 (9.4-10.5) | 2,535 | 14.3 (13.4-15.3) | 734 | 5.1 (4.5-5.7) | <.0001 |

N in each cell represents unweighted frequency or numerator and N on top row represents denominator. % is prevalence weighted for sampling and non-response.

AI/AN = American Indian/Alaska Native

PI = Pacific Islander

^a^Any underlying conditions includes a condition from one of the following major underlying condition categories (see below)

^b^Major underlying conditions include: chronic lung disease including asthma; chronic metabolic disease including diabetes; blood disorders/hemoglobinopathies; cardiovascular disease (excluding hypertension); neurologic disorder; immunocompromised condition; renal disease; any obesity; postpartum; gastrointestinal or liver disease; rheumatologic, autoimmune, or inflammatory conditions; other conditions. For definition of major conditions, see Supplemental Table 4.

^c^Cardiovascular disease excludes hypertension.

^d^Obesity is defined as calculated body mass index (BMI) ≥30 kg/m2, and if BMI is missing, by International Classification of Diseases discharge diagnosis codes.

^e^Pre-Delta: March 2020**–**June 2021, Delta: July 2021**–**December 18,2021, Omicron: December 19, 2021**–**April 2022

^f^Long-term care facility residence includes nursing home/skilled nursing facility, alcohol/drug abuse treatment center, other rehabilitation facility, assisted living/residential care, group/retirement homes, long-term care facility (LTCF), long term acute care hospital (LTACH), or any other psychiatric facility.

**Table S2:** Adjusted relative risk for intensive care among hospitalized adults with COVID-19 who had bacterial testing performed within 7 days of admission, COVID-NET March 2020**–**April 2022

|  | Adjusted relative risk for intensive Care (95% CI) n=16,373 | p-value |
| --- | --- | --- |
| Bacterial infection in a respiratory or sterile site within 7 days of hospital admission | 2.11 (1.95, 2.23) | <.0001 |
| Sex |  |  |
| Female | Ref | Ref |
| Male | 1.21 (1.15, 1.28) | <.0001 |
| Age category |  |  |
| 18-34 years | Ref | Ref |
| 35-54 years | 1.04 (0.9, 1.21) | 0.5969 |
| 55-74 years | 1.26 (1.12, 1.42) | 0.0001 |
| ≥75 years | 1.04 (0.8, 1.34) | 0.769 |
| Race/Ethnicity |  |  |
| Non-Hispanic White | Ref | Ref |
| Non-Hispanic Black | 1.14 (1.00, 1.30) | 0.056 |
| Non-Hispanic AI/AN | 1.1 (0.91, 1.32) | 0.323 |
| Asian/PI | 1.26 (1.12, 1.42) | <0.0001 |
| Hispanic | 1.27 (1.14, 1.43) | 0.0016 |
| Chronic lung disease | 1.09 (1.01, 1.17) | 0.0298 |
| Diabetes | 1.20 (1.1, 1.31) | <0.0001 |
| Cardiovascular disease | 0.98 (0.89, 1.09) | 0.7563 |
| Obesity | 1.11 (1.01, 1.21) | 0.0264 |
| GI/liver disease | 1.06 (0.87, 1.28) | 0.5776 |
| Renal disease | 1.08 (1.00, 1.17) | 0.0518 |
| Time period^a^ |  |  |
| Pre-delta | Ref | Ref |
| Delta | 1.16 (1.01, 1.33) | 0.0305 |
| Omicron | 0.82 (0.64, 1.06) | 0.136 |

AI/AN = American Indian/Alaska Native

PI = Pacific Islander

^a^Pre-Delta: March 2020**–**June 2021, Delta: July 2021**–**December 18,2021, Omicron: December 19, 2021**–**April 2022

**Table S3:** Adjusted relative risk for mechanical ventilation among hospitalized adults with COVID-19 who had bacterial testing performed within 7 days of admission, COVID-NET March 2020**–**April 2022

|  | Adjusted relative risk for mechanical ventilation (95% CI) n=16,373 | p-value |
| --- | --- | --- |
| Bacterial infection in a respiratory or sterile site within 7 days of hospital admission | 3.04 (2.74, 3.37) | <.0001 |
| Sex |  |  |
| Female | Ref | Ref |
| Male | 1.31 (1.15, 1.49) | <0.0001 |
| Age category |  |  |
| 18-34 years | Ref | Ref |
| 35-54 years | 1.31 (1.11, 1.53) | 0.0011 |
| 55-74 years | 1.64 (1.37, 1.96) | <.0001 |
| ≥75 years | 1.29 (0.98, 1.7) | 0.0687 |
| Race/Ethnicity |  |  |
| Non-Hispanic White | Ref | Ref |
| Non-Hispanic Black | 1.22 (1.14, 1.31) | <.0001 |
| Non-Hispanic AI/AN | 1.09 (0.83, 1.43) | 0.5378 |
| Asian/PI | 1.54 (1.36, 1.74) | <.0001 |
| Hispanic | 1.52 (1.29, 1.78) | <.0001 |
| Chronic lung disease | 1.19 (1.06, 1.33) | 0.0027 |
| Diabetes | 1.16 (1.01, 1.34) | 0.031 |
| Cardiovascular disease | 0.9 (0.81, 0.99) | 0.0339 |
| Obesity | 1.27 (1.15, 1.4) | <.0001 |
| Gastrointestinal/liver disease | 1.13 (0.89, 1.42) | 0.3215 |
| Renal disease | 1.07 (0.92, 1.25) | 0.3869 |
| Time period^a^ |  |  |
| Pre-delta | Ref | Ref |
| Delta | 1.15 (1.06, 1.25) | 0.0008 |
| Omicron | 0.60 (0.45, 0.78) | 0.0002 |

AI/AN = American Indian/Alaska Native

PI = Pacific Islander

^a^Pre-Delta: March 2020**–**June 2021, Delta: July 2021**–**December 18,2021, Omicron: December 19, 2021**–**April 2022

**Table S4:** Underlying Condition Categories

| **Major Underlying Condition Category** | **Specific Conditions Included** |
| --- | --- |
| Chronic Lung Disease | 1. Active Tuberculosis (TB) 2. Asbestosis 3. Asthma/Reactive airway disease (RAD) 4. Bronchiectasis 5. Bronchiolitis Obliterans 6. Chronic bronchitis 7. Chronic respiratory failure 8. Cystic fibrosis (CF) 9. Emphysema/ Chronic Obstructive Pulmonary Disease (COPD) 10. Interstitial Lung Disease (ILD) 11. Obstructive Sleep Apnea 12. Oxygen (O_2_) dependent 13. Pulmonary Fibrosis 14. Restrictive Lung Disease 15. Sarcoidosis |
| Chronic Metabolic Disease | Adrenal Disorders (Addison’s, Adrenal Insufficiency, Cushing Syndrome, Congenital Adrenal Hyperplasia)  Diabetes Mellitus (DM)  Glycogen or other storage diseases  Hyper/Hypo function of pituitary gland  Inborn errors of metabolism  Metabolic Syndrome  Parathyroid Dysfunction (Hyperparathyroidism, Hypoparathyroidism)  Thyroid dysfunction (Grave’s disease, Hashimoto’s Disease, Hyperthyroidism, Hypothyroidism) |
| Blood Disorders/  Hemoglobinopathies | Alpha Thalassemia  Aplastic anemia  Coagulopathy (Factor V Leiden, Von Willebrand Disease)  Hemoglobin S-beta thalassemia  Leukopenia  Myelodysplastic syndrome  Neutropenia  Pancytopenia  Polycythemia vera  Sickle cell disease  Splenectomy/Asplenia  Thrombocytopenia |
| Cardiovascular Disease | Aortic aneurysm (AAA), history of  Aortic/Mitral/Tricuspid/Pulmonic valve replacement, history of  Aortic Regurgitation (AR)  Aortic stenosis (AS)  Atherosclerotic cardiovascular disease (ASCVD)  Atrial fibrillation (A-fib)  Atrioventricular (AV) blocks  Automated implantable devices (AID/AICD)/Pacemaker  Bundle Branch Block (BBB/RBBB/LBBB)  Cardiomyopathy  Carotid Stenosis  Cerebral vascular accident (CVA)/ incident/Stroke, history of  Congenital heart disease (specify)  Atrial septal defect  Pulmonic stenosis  Tetralogy of Fallot  Ventricular septal defect  Other, specify  Coronary artery bypass grafting (CABG), history of  Coronary artery disease (CAD)  Deep vein thrombosis (DVT), history of  Heart Failure/Congestive heart failure (CHF)  Myocardial infarction (MI), history of  Mitral regurgitation (MR)  Mitral stenosis (MS)  Peripheral artery disease (PAD)  Peripheral vascular disease (PVD)  Pulmonary embolism (PE), history of  Pulmonary hypertension (PHTN)  Pulmonic regurgitation  Pulmonic stenosis  Transient ischemic attack (TIA), history of  Tricuspid regurgitation (TR)  Tricuspid stenosis  Ventricular fibrillation (VF, VFIB), history of  Ventricular tachycardia (VT, VTACH), history of  Valvular disorder not otherwise specified |
| Neurologic Disorder | Amyotrophic lateral sclerosis (ALS)  Cerebral palsy  Cognitive dysfunction  Dementia/Alzheimer’s disease  Developmental delay  Down syndrome/Trisomy 21  Edward’s syndrome/Trisomy 18  Epilepsy/seizure/seizure disorder  Mitochondrial disorder  Multiple sclerosis (MS)  Muscular dystrophy  Myasthenia gravis (MG)  Neural tube defects/Spina bifida  Neuropathy  Parkinson’s disease  Plegias/Paralysis/Quadriplegia  Scoliosis/Kyphoscoliosis  Traumatic brain injury (TBI), history of |
| Immunocompromised Conditions | AIDS or CD4 count <200  Complement Deficiency  HIV Infection  Immunoglobulin Deficiency/Immunodeficiency  Immunosuppressive Therapy (within the last 12 months prior to admission)  If yes, for what condition?  Leukemia*  Lymphoma/Hodgkins/Non-Hodgkins (NHL)*  Metastatic cancer*  Multiple Myeloma*  Solid organ malignancy*  If yes, which organ?  Steroid Therapy (within 2 weeks of admission)  Transplant, hematopoietic stem cell (Bone marrow transplant (BMT), peripheral stem cell transplant (PSCT)), history of  Transplant, Solid Organ (SOT), history of |
| Renal Disease | Chronic kidney disease/chronic renal insufficiency (CRI)  End stage renal disease (ESRD)  Dialysis (HD)  Glomerulonephritis (GN)  Nephrotic syndrome  Polycystic Kidney Disease (PCKD) |
| Any obesity | Obese  Severely/morbidly obese |
| Post-partum (two weeks or less) |  |
| Gastrointestinal/ Liver Disease | Alcoholic hepatitis  Autoimmune hepatitis  Barrett’s esophagitis  Chronic liver disease  Cirrhosis/End stage liver disease (ESLD)  Crohn’s disease  Esophageal varices  Esophageal strictures  Hepatitis B, chronic (HBV)  Hepatitis C, chronic (HCV)  Non-alcoholic fatty liver disease (NAFLD)/NASH  Ulcerative colitis (UC) |
| Rheumatologic/Autoimmune/Inflammatory Conditions | Ankylosing Spondylitis  Dermatomyositis  Juvenile Idiopathic Arthritis  Kawasaki disease  Microscopic polyangiitis  Polyarteritis nodosum (PAN)  Polymyalgia Rheumatica  Polymyositis  Psoriatic Arthritis  Rheumatoid Arthritis (RA)  Systemic Lupus Erythematosus (SLE)/Lupus  Systemic Sclerosis  Takayasu arteritis  Temporal/Giant Cell Arteritis  Vasculitis, other |
| Other | Hypertension (HTN)  Feeding tube dependent  Trach-dependent/Vent dependent  Wheelchair dependent  Other, specify |

**Figure S1:** Percent of sampled cases with bacterial culture testing done quarterly over time, COVID-NET March 2020**–**April 2022
